# The biomarkers and potential pathogenesis of lung cancer related cerebral hemorrhage

**DOI:** 10.1097/MD.0000000000015693

**Published:** 2019-05-17

**Authors:** Kemin Qin, Yicong Chen, Haiyin Long, Jiyun Chen, Dacheng Wang, Li Chen, Zhijian Liang

**Affiliations:** a Department of Neurology, The First Affiliated Hospital of Guangxi Medical University, Nanning City, Guangxi; b Department of Neurology, The First Affiliated Hospital of Sun Yat-sen University, Guangzhou City, Guangdong; c Department of Neurology, The Ninth Affiliated Hospital of Guangxi Medical University, Beihai City, Guangxi, PR China.

**Keywords:** biomarkers, cerebral hemorrhage, index, lung cancer, pathogenesis

## Abstract

Cerebral hemorrhage is one of the common complications in patients with lung cancer (LC). Although cancer related cerebral hemorrhage was aware, the pathogenesis and biomarkers of lung cancer related cerebral hemorrhage (LCRCH) remained not well known. The aim of this study was to investigate the pathogenesis and plasma biomarkers of LCRCH.

A retrospective review was conducted on acute cerebral hemorrhage patients with active LC who was admitted to the hospital between January 2007 and December 2017. A total of 56 patients with LCRCH (active LC patients with acute cerebral hemorrhage but without conventional vascular risks) was recruited. Meanwhile, 112 patients with active LC alone and gender, age, and subtype of cancer cell matched were recruited as control group.

In LCRCH patients, most of the hemorrhagic lesions were located in lobes. And most of them with adenocarcinoma were in medium to terminal stage with poor prognosis short-term. Moreover, LCRCH patients had a lengthened prothrombin time (PT), elevated plasma carcinoembryonic antigen (CEA), cancer antigen 125 (CA125) and cancer antigen 199 (CA199) levels and decreased platelet (PLT) level than did the patients with LC. Multivariate logistic regression analysis showed that lengthened PT, elevated plasm CEA, and CA199 levels were independent risk factors for LCRCH.

It was suggested that lengthened PT, elevated plasm CEA and CA199 levels associated with the pathogenesis of LCRCH, and that the Index derived from independent risks should be serve as a specific biomarker of LCRCH.

## 1 Introduction

Cerebral hemorrhage is a common complication of patients with cancer,^[1,2]^ and the risks for cerebral hemorrhage increase in patients with cancer,^[3,4]^ indicating that cancer itself can directly or indirectly lead to the development of cerebral hemorrhage. As cancer was found involved in the ischemic stroke and which was regarded as cancer related ischemic stroke,^[5–7]^ the cerebral hemorrhage caused directly or indirectly by cancer could be regarded as cancer related cerebral hemorrhage. Previous studies conducted on general cancer patients showed that the common characteristics of cancer related cerebral hemorrhage in most patients were coagulant dysfunction but without conventional risks for stroke.^[8,9]^ Moreover, previous studies also revealed that cancer related cerebral hemorrhage was caused directly by intratumoral bleeding in primary brain cancer or intracranial metastasis from systemic cancer, directly by cancer related coagulant dysfunction, and indirectly by the adverse effects of cancer related therapies.^[8–10]^ Nevertheless, cancer has various subtypes in terms of cell types, growth zones and growing status, which may lead to cancer related cerebral hemorrhage by various pathogenesis. As a result, up to now, an exact clinical feather and the precise pathogenesis of cancer related cerebral hemorrhage remain to be illuminated. Therefore, it is reasonable that the study focal on a certain type of cancer may be more conducive to illuminate of the pathogenesis about cancer related cerebral hemorrhage.

LC is the most common malignancy and the most frequent cause of cancer mortality in the world. In 2012, there were 1.82 million people suffered from LC and 1.59 million deaths of LC worldwide, respectively.^[11]^ And it is estimated that more than 1 out of every 3 LCs occurred in China.^[12]^ The incidence of LC is high in the south of China. There were 80.5 and 68.8 thousand new cases and deaths of LC at these regions in 2015.^[13]^ At the same time, LC has become the leading cause of cancer-related deaths in these areas and seriously endangered the health of local people.^[13]^ Moreover, it is noteworthy that LC is one of the most frequent malignancies combined with cerebral hemorrhage.^[2,9]^ And recent studies showed that the risks of cerebral hemorrhage were increased in LC patients compared to normal population,^[4,14]^ suggesting that LCRCH existed theoretically. Furthermore, it was reported that some cerebral hemorrhage patients with conceal LC without conventional risk factors but smoking for cerebral hemorrhage,^[9,15,16]^ indicating LCRCH not only existed but may do harm to the body anterior to the direct impacts of LC. However, it is still very difficult to identify LCRCH from cerebral hemorrhage caused by other pathogens in contemporary clinical practice. Therefore, it is very important to recognize the biomarkers and the pathogenesis of LCRCH. In present study, our aim is to investigate the biomarkers and potential pathogenesis of LCRCH.

## 2 Materials and methods

### 2.1 Patient selection

This study was reviewed and approved by the Guangxi Medical University Review Board. LC patients were recruited from the First Affiliated Hospital of Guangxi Medical University, the Ninth Affiliated Hospital of Guangxi Medical University and the First Affiliated Hospital of Sun Yat-Sen University between January 2007 and December 2017. The diagnosis of LC was established by histopathological examination and the judge of the stages of LC was referred to the stage classification of LC.^[17]^ Referring the definition of active cancer in the study of Lee and his leagues,^[18]^ active LC in present study was defined as a diagnosis of LC within 6 months before enrollment, any treatment for LC within the previous 6 months, or recurrent or metastatic LC. The diagnostic of cerebral hemorrhage was referred to the 2015 Guidelines for the Diagnosis and Treatment from the American Heart Association.^[19]^

In actuality, it is very difficult to identify LCRCH in contemporary clinical practice. Referred to the definition of cancer related stroke in several researches on cancer related ischemic stroke,^[5–7]^ LCRCH in the present study was defined as the cerebral hemorrhage developed in patients with active LC without conventional vascular risk factors including hypertension, atrial fibrillation, diabetes, and intracranial arterial aneurysm. The LCRCH group inclusion criteria as follows:

1. Active LC patients were admitted to the hospital with sudden-onset of slurring speech, paralysis and numbness of limbs, or other focal neurological deficits. Hemorrhagic lesions were confirmed by brain computed tomography (CT) or magnetic resonance imaging (MRI), and All LCRCH patients underwent CT or MRI scans within 7 days to confirm cerebral hemorrhage, and within the 28 days after symptom onset underwent at least 1 time of enhanced CT or MRI scans to confirm cerebral hemorrhage again and to rule out intracranial metastasis.
2. Patients hospitalized with acute cerebral hemorrhage were firstly confirmed with LC.
3. All patients were evaluated by 3 independent experts from oncology, neurology, and neuro-imaging, who were not otherwise associated with the planning and conduct of the study.

And the exclusion criteria as follows:

1. presence of conventional vascular risk factors or
2. LC diagnosed >5 years ago, with no evidence of recurrence or metastasis;
3. presence of cerebral infarction and the other diseases in central never system;
4. presence of anticoagulant therapy or renal and hepatic insufficiency.

**Figure 1:**
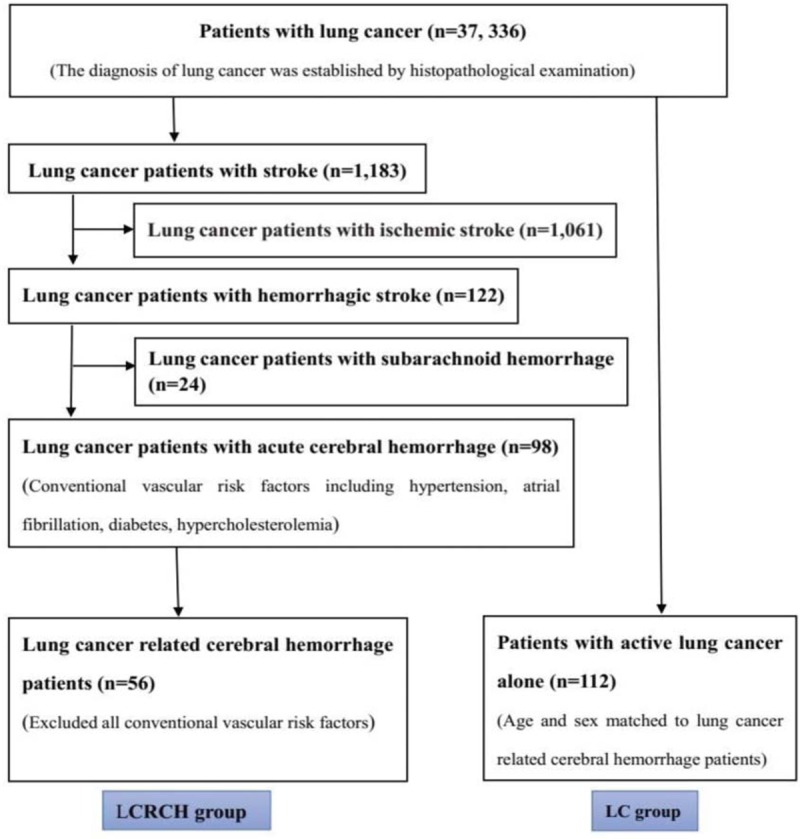
Patient selection. Fifty-six active lung cancer related cerebral patients were recruited as lung cancer related cerebral hemorrhage (LCRCH) group. Meanwhile, 112 patients with active lung cancer alone were recruited as control.

Patients with active LC alone group recruited active LC patients whose gender, age, and subtype of cancer matched to LCRCH patients without cerebral hemorrhage. The exclusion criteria were similar to the LCRCH group patients (Fig. 1).

### 2.2 Collection of clinical materials

**Figure 2:**
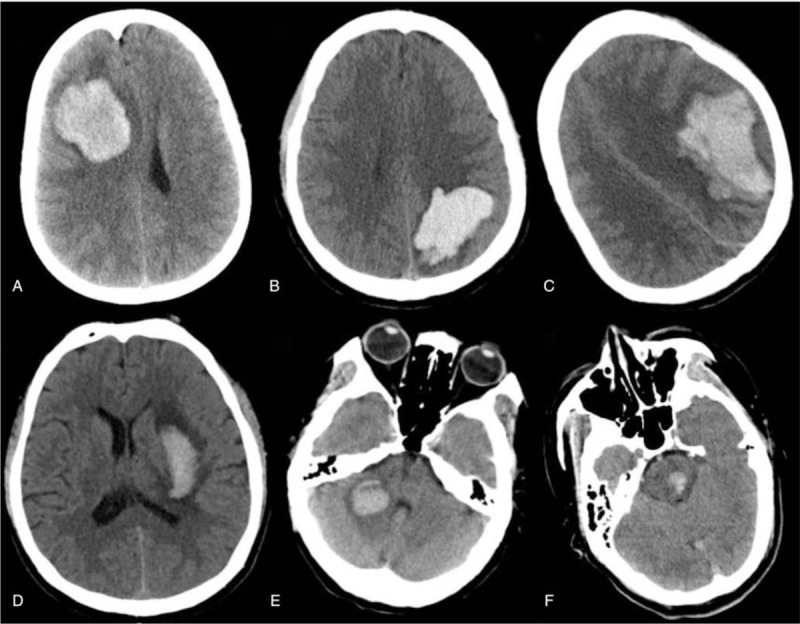
CT images came from 6 active lung patients without conventional vascular risk factors for cerebral hemorrhage. Pictures A–F were brain CT axial view, and the locations of cerebral hemorrhage were shown on the right frontal lobe, left parietal lobe, left frontal parietal temporal lobe, left basal ganglia, right cerebellar hemisphere, and brain stem respectively. It was showed that the lobar hemorrhage was greater than that in other parts of the brain from the above pictures. CT = computed tomography.

The general demographic data in both groups, such as age and gender, were collected. Moreover, data were collected from blood tests, including routine blood tests and biochemical blood examinations, coagulation indexes, cancer biochemical markers. Data on LC were collected, including the clinical manifestations; pathological types; tumor node metastasis (TNM) stages of cancer, and the therapies for cancer (such as surgical resection, chemotherapy, and radiotherapy). The information related to acute cerebral hemorrhage was collected, such as clinical symptoms and signs, severity of neurological functions, hemorrhagic locations, and therapeutic measure. The National Institute of Health Stroke Scale (NIHSS) was used for evaluating the severity of focal neurological deficit. And other data including electrocardiography (ECG), cardiac color Doppler ultrasound, cervical blood Doppler, brain CT, and CT angiography (CTA), brain magnetic resonance image (MRI) and magnetic resonance angiography (MRA), were also collected. The prognosis of patients at the 30th day after hemorrhage onset was assessed with the modified Rankin scale (mRS), and mRS scores >3 indicated the poor prognosis (Fig. 2).

### 2.3 Statistical methods

All statistical analyses were performed using SPSS 20.0 software. Independent-samples *t* tests were used for quantitative data. Frequencies of normally distributed variables were compared using *χ*^2^or Fisher exact tests. Multivariable logistic regression analysis was conducted to predict the independent contributions of factors in LCRCH versus LC groups. Significant variables with *P* < .10 in univariate analyses were considered explanatory variables and were entered together into multivariable models. A *P* value <.05 was examined statistically significant. The variables with *P* < .05 in condition multivariate analysis were regarded as the independent risk factors for cerebral hemorrhage. Considering the independent risk factors may together trigger the development of cerebral hemorrhage in LC patients, we calculate the Index of LC related cerebral hemorrhage (details in results section).

## 3 Results

In present study, out of 37,336 LC patients, 56 patients (0.15%) met the criteria of LCRCH were recruited. Meanwhile, 112 patients with active LC alone were recruited as control. As a result of selection, there were 60.7% male and 39.3% female in the 2 groups. The age of LCRCH patients (59.15 ± 13.49) years and LC patients (58.73 ± 13.66) was not significantly different (*P* > .05) (Table 1).

**Table 1:**
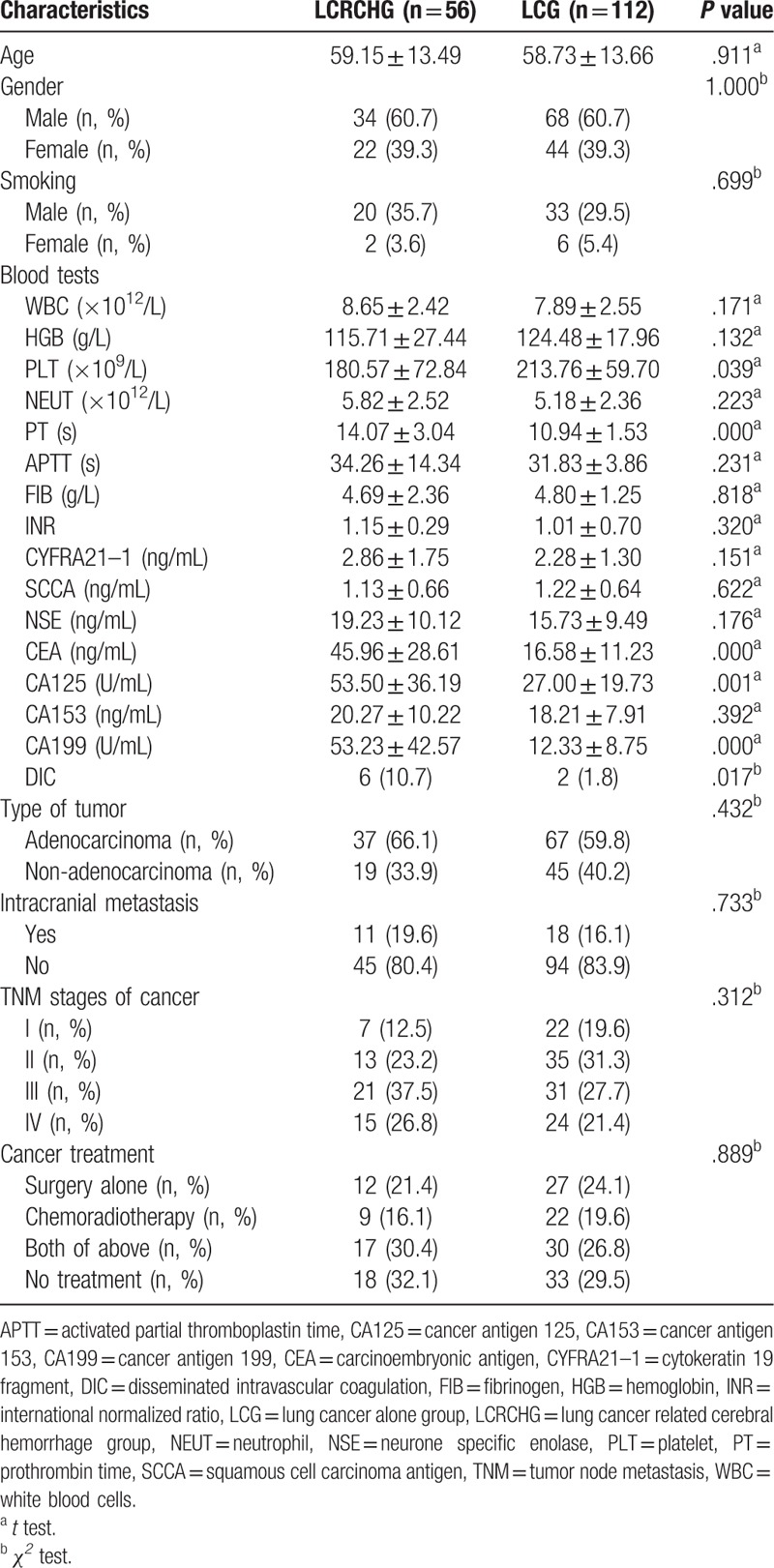
The clinical features of 2 groups patients.

There were 12 (21.4%) patients who were firstly hospitalized with cerebral hemorrhage and were firstly confirmed to have LC during the therapy anti-stroke. Among other LCRCH patients, developed cerebral hemorrhage following the diagnosis of LC, there were 17 (30.4%) patients within 6 months, 17 (30.4%) between 7 and 12 months, 10 (17.8%) patients between 1 and 2 years after diagnosis. All of LCRCH patients had some degree of neurological deficits, and the mean NIHSS score was 11.32 ± 7.37 (range: 2–28) at the day of cerebral hemorrhage onset. Most patients had hemorrhagic lesions in the lobes (29, 51.8%), conservative treatment (32, 57.1%), and with poor prognosis (34, 60.7%) at 30 days after cerebral hemorrhage occurred (Table 2).

**Table 2:**
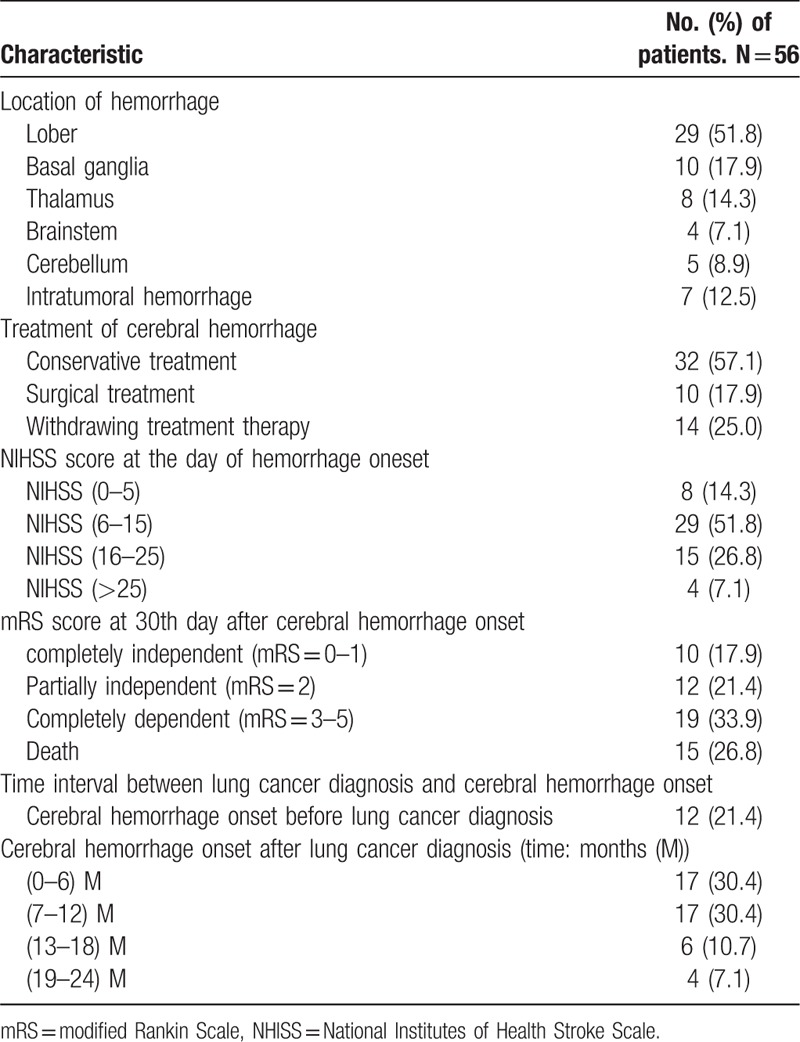
Data about cerebral hemorrhage.

As a consequence of the inclusion and exclusion criteria, there was no significant difference in the types of LC in the LCRCH and LC patients. At the same time, the clinical characteristics between the 2 groups, such as most items of blood routine, staging of LC, and cancer therapies were not significantly different. However, LCRCH patients had a lengthened PT, elevated plasma CEA, CA125, and CA199 levels and decreased PLT level than did the LC patients (Table 1).

To identify the potential risk factors for LCRCH, multivariate logistic regression analysis was utilized to investigate 5 potentially important variables: PT, CEA, CA125, CA199, and PLT. As a result of multivariate logistic regression analysis, it was revealed that prolonged PT, elevated plasma CEA and CA199 levels may be the independent risk factors for LCRCH. And the risk of cerebral hemorrhage in patients with LC increased by 77.1% (odds ratio [OR] 2.161; 95% confidence interval [CI] 1.349, 3.464; *P* = .001) with a lengthened in PT level of per second. The risk of cerebral hemorrhage increased by 5.9% (OR 1.060; 95% CI 1.008, 1.115; *P* = .022) with an increase in CEA of 1 ng/mL, and by 7.3% (OR 1.076; 95% CI 1.008, 1.128; *P* = .003) with an increase in CA199 of 1 U/mL. (Table 3)

**Table 3:**
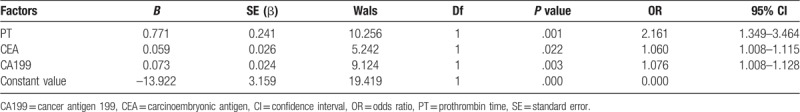
Multivariate logistic regression analysis of independent predictors of cerebral hemorrhage risk in lung cancer patients.

Furthermore, to investigate the specific biomarkers of LC related cerebral hemorrhage, the Index of LC related cerebral hemorrhage was calculated as follows: firstly, the products of PT, CEA, and CA199 from both LCRCH group (35,786.35 ± 32,323.90) and LC group (2424.96 ± 2263.57) were calculated. And the products of PT, CEA, and CA199 from LCRCH groups were larger compared to that of LC groups (*P* < .05). Secondly, the 95% confidence interval (CI) of the mean value of the product of PT, CEA, and CA199 from LCRCH group was calculated with [22,730.45, 48,842.26]. Finally, the lower bound of 95% CI (22,730.45) was regarded as the Index of LC related cerebral hemorrhage.

## 4 Discussion

Cerebral hemorrhage is one of the complications of the central nervous system in patients with cancer.^[1,2]^ In 2012, to investigate the incidence of cerebral hemorrhage in cancer patients, Zöller et al^[3]^ retrospectively analyzed the medical data of 820,491 patients registered in the Sweden's National MigMed 2 Database from 1987 to 2008 and found that the incidence of cerebral hemorrhage after cancer diagnosis within 6 months, 6 months to 1 year, and 1 year to 5 years was 2.2%, 1.4%, and 1.3%, respectively, which were significantly higher than the incidence of the population without cancer. Which indicated that cancer could increase the risks for cerebral hemorrhage and cancer related cerebral hemorrhage theoretically existed. Moreover, the incidence of cerebral hemorrhage was found increased in patients with LC, indicating LCRCH theoretically existed too.^[4,14]^ As it was very difficult to identify LCRCH in modern clinical practice, referred to the definition of cancer related stroke in several researches on cancer related ischemic stroke,^[5–7]^ LCRCH in present study was defined as the cerebral hemorrhage developed in patients with active LC without conventional vascular risk factors. It was found that the most LCRCH patients developed cerebral hemorrhage within 12 months after the diagnosis of LC, indicating that in terms of time internal between LC diagnosis and the cerebral hemorrhage occurrence, LCRCH had the similar feather as the general cancer related cerebral hemorrhage, and that as soon as LC was diagnosed, some measures should be taken to prevent cerebral hemorrhage. As cancer patients sometimes presented cerebral hemorrhage as the initial manifestation,^[15,20–22]^ some LC patients were firstly hospitalized for cerebral hemorrhage and were confirmed to have occult LC in present study. It was suggested that such patients should be received more attention especially for unexplained cerebral hemorrhage. The characteristics of coagulant dysfunction such as prolonged PT, elevated plasma CEA, and CA199 levels in present study may be helpful to identify occult LC in patients with acute cerebral hemorrhage.

The pathogenesis of cancer leading to cerebral hemorrhage had received attention for a long time. In order to identify the pathogenesis of cancer related cerebral hemorrhage, Zhang and his colleagues^[23]^ retrospectively reviewed 1667 patients hospitalized in Bankstown-Lidcombe Hospital in Australia from January 1999 to December 2004 and found that the PT and activated partial thromboplastin time (APTT) were prolonged in cerebral hemorrhage patients with cancer than that in non-cancer patients, indicating that coagulation dysfunction may involve the cerebral hemorrhage in cancer patients. Moreover, Navi et al^[9]^ analyzed 141 systemic cancer patients with cerebral hemorrhage registered in Memorial Sloan-Kettering cancer center from 2000 to 2007 and found that 98 (69.50%) patients developed cerebral hemorrhage due to brain metastasis, 55 (39.01%) patients due to coagulation dysfunctions, and 33 (23.40%) patients due to both intratumoral hemorrhage and coagulation dysfunction, indicating that both intratumoral hemorrhage of intracranial metastasis and coagulation dysfunction were the major pathogenesis of cancer related cerebral hemorrhage. In present study, a small part of LCRCH patients was found to have intratumoral hemorrhage, suggesting that intratumoral hemorrhage was also one of the pathogenesis of LC related cerebral hemorrhage. However, most of LCRCH patients were found to have a lengthened PT, and further analyzed showed that the lengthened PT was the independent risk factor for cerebral hemorrhage in present study, suggesting that coagulant dysfunction played a crucial role in pathogenesis on LCRCH. Moreover, previous research also found that cerebral hemorrhage in cancer patients was closely related to platelet (PLT) counts decline.^[10,24–27]^ In present study, the PLT counts in LCRCH patients were significantly declined compared to that in LC patients, indicating that PLT counts decline may be another one of the pathogenesis on LC related cerebral hemorrhage. Furthermore, disseminated intravascular coagulation (DIC), a sever coagulant dysfunction statues, was found to cause cerebral hemorrhage in patients with cancer, including patients with LC.^[22,28–30]^ In present study, 6 LCRCH patients were found with DIC, suggesting that DIC gave rise to cerebral hemorrhage in patients with LC again. It was suggested that coagulation dysfunction was the major pathogenesis of LCRCH.

In addition, nonbacterial thrombotic endocarditis (NBTE) in LC patients had been found led to cerebral hemorrhage in previous researches.^[31,32]^ Radiotherapy and chemotherapy in previous researches, such as whole brain irradiation and several agents including gefitinib, bevacizumab, and sunitinib might aggravate coagulation dysfunctions and lead to cerebral hemorrhage in patients with LC.^[33–35]^ Although such causes leading cerebral hemorrhage mentioned above were not found in present study, they deserved the attention from clinicians.

However, it was unclear that by what way cancer caused coagulation dysfunctions. Previous studies found that the elevated plasma cancer markers (CA125, CA153, CA199, CEA, and alpha-fetoprotein) may associate with cerebral hemorrhage patients with cancer.^[15,36]^ In present study, it was found that elevated plasma CEA and CA199 levels were the independent risks for cerebral hemorrhage in patients with active LC. Although it was still unclear, it was interesting that the elevated CEA and CA199 levels play what a role in the pathogenesis of LCRCH. As it was found that elevated plasma CA125 level was related to the development of ischemic stroke in patients with cancer in previous studies.^[37,38]^ As it was determined that carcinoma mucin secreted by cancer cells triggered the reciprocal activation of PLTs and neutrophils, leading to the formation of multiple microthromboembolism in the blood by animal experiments.^[39]^ In this way, as plasma cancer marker, including CA125, CA199, and CEA were also carcinoma mucin secreted by cancer cells, it may be speculated that the elevated plasma cancer marker levels could lead to PLT activation. In early stay of cancer, the elevated plasma cancer marker levels, including CA125, CA199, and CEA, could lead to PLTs increase in blood and hypercoagulable state, which led to ischemic stroke finally. However, in later stay, owing to the consumption, the elevated plasma cancer marker levels could lead to PLTs decrease and coagulation dysfunctions, which lead to cerebral hemorrhage finally. However, the pathogenesis about elevated plasma cancer marker levels leading to LCRCH needed further researches in future.

Considering the lengthened PT, elevated plasma CEA, and CA199 levels were found as independent risks for cerebral hemorrhage, respectively, and the independent risks may play a role in cerebral hemorrhage with active LC together. The Index of LCRCH derived from the mean value of products of PT, plasma CEA and CA199 levels in LCRCH patients, indicating that when the products of PT, the plasma CEA, and CA199 levels rise and reach at a point (such as the Index) the LCRCH should be triggered. The Index of LCRCH, more or less, should be used as a specific biomarker to identify the LCRCH from various subtypes of cerebral hemorrhage caused by other etiology.

## 5 Conclusions

In summary, it was suggested that lengthened PT, elevated plasma CEA, and CA199 levels associated with the pathogenesis of LCRCH, and that the Index derived from independent risks should be used a specific biomarker of LCRCH.

## Author contributions

**Conceptualization:** Kemin Qin, Yicong Chen, Dacheng Wang, Zhijian Liang.

**Data curation:** Kemin Qin, Yicong Chen, Haiyin Long, Jiyun Chen, Dacheng Wang, Zhijian Liang.

**Formal analysis:** Kemin Qin, Yicong Chen, Haiyin Long, Jiyun Chen, Li Chen.

**Funding acquisition:** Zhijian Liang.

**Investigation:** Kemin Qin, Yicong Chen, Haiyin Long, Jiyun Chen.

**Methodology:** Kemin Qin, Yicong Chen, Haiyin Long, Jiyun Chen, Li Chen, Zhijian Liang.

**Project administration:** Zhijian Liang.

**Resources:** Kemin Qin, Yicong Chen, Dacheng Wang.

**Software:** Kemin Qin, Yicong Chen, Zhijian Liang.

**Supervision:** Dacheng Wang.

**Validation:** Li Chen.

**Visualization:** Zhijian Liang.

**Writing – original draft:** Kemin Qin.
